# Synergistic effects of climate and urbanisation on the diet of a globally near threatened subtropical falcon

**DOI:** 10.1002/ece3.70290

**Published:** 2024-09-10

**Authors:** Mohammod Foysal, Connor T. Panter

**Affiliations:** ^1^ Independent Raptor Researcher Dhaka Bangladesh; ^2^ School of Geography University of Nottingham Nottingham UK; ^3^ School of Applied Sciences University of Brighton Brighton UK

**Keywords:** Bangladesh, climate change, *Falco chicquera*, raptor, red‐necked falcon, trophic ecology

## Abstract

Understanding how human activities affect wildlife is fundamental for global biodiversity conservation. Ongoing land use change and human‐induced climate change, compel species to adapt their behaviour in response to shifts in their natural environments. Such responses include changes to a species' diet or trophic ecology, with implications for the wider ecosystem. This is particularly the case for predatory species or those that occupy high positions within trophic webs, such as raptors. Between 2002 and 2019, we observed 1578 feeding events of the globally near threatened and understudied, Red‐necked Falcon (*Falco chicquera*) in Bangladesh. We explored the effects of mean monthly temperature, precipitation, temperature differences, and urban land cover on (a) mean prey weights and (b) dietary composition of 15 falcon pairs. Falcons hunted smaller prey items during months with increased temperatures and precipitation, and in more urban areas. However, during months with increased temperature differences, falcons tended to prey on larger prey items. Being specialist aerial hunters, these dietary patterns were largely driven by the probabilities of bats and birds in the diet. Falcons were more likely to prey on bats during warmer and wetter months. Furthermore, urban pairs tended to prey on bats, whereas more rural pairs tended to prey on birds. Mean monthly temperature difference, i.e., a proxy for climate change, was better at explaining the probability of bats in the falcon diet than mean monthly temperature alone. Anthropogenic dietary shifts can have deleterious effects on species with declining populations or those of conservation concern. The effects of urbanisation and human‐induced climate change are expected to continue into the foreseeable future. Therefore, our findings represent a cornerstone in our understanding of how falcons respond to an increasingly human‐dominated world.

## Graphical abstract

Understanding how human activities affect wildlife is fundamental for global biodiversity conservation. We explored the effects of climate and urbanisation on average prey weight and dietary composition of the globally near threatened Red‐necked Falcon in Bangladesh.

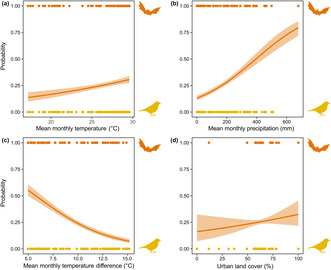

## 1 INTRODUCTION

Advancing our understanding of how human activities affect wildlife is a fundamental objective of global biodiversity conservation (Rands et al., 2010; Robinson, 2006; Taylor‐Brown et al., 2019). Anthropogenic activities, such as ongoing land use change and human‐induced climate change, compel species to adapt their behaviour in response to shifts in their natural environments (Buchholz et al., 2019; Lowry et al., 2012). Adapting to increasingly human‐dominated landscapes exerts changes to a species' behavioural ecology including shifts in migration patterns (Kavwele et al., 2022), flight initiation distance (Hall et al., 2020), reproductive success (Sumasgutner et al., 2019) and dietary composition (Birnie‐Gauvin et al., 2017; Restrepo‐Cardona et al., 2024).

Dietary changes, in response to increasing human pressures, can have deleterious effects throughout the wider ecosystem (Robb et al., 2008). This is particularly the case for predatory species or those that occupy high positions within trophic webs, such as raptors (Demerdzhiev et al., 2023). Raptors, i.e., Accipitriformes, Cathartiformes, Falconiformes, Strigiformes and Cariamiformes (McClure et al., 2019), are sensitive to the impacts of human activities and have been regarded as sentinels of the wider ecosystem (Donázar et al., 2016; Grande et al., 2018). Land use change and human‐induced climate change can lead to population declines in key prey species (Sidiropoulos et al., 2022) or even result in an overabundance of particular prey (Costán & Sarasola, 2021). Changes to a species' trophic ecology can impact its' wider population, with negative impacts on species experiencing population declines or those of conservation concern, such as the Red‐necked Falcon (*Falco chicquera*).

The Red‐necked Falcon is a small‐sized raptor in the family Falconidae (del Hoyo et al., 2020). The species occurs in two disjunct populations distributed across Africa and the Indian subcontinent (del Hoyo et al., 2020). Within the two disjunct populations, three subspecies have been described (Africa: *F. chicquera ruficollis* and *F. c. horsbrughi*; Asia: *F. c. chicquera*) with some authorities considering the African and Asian populations completely separate (Bhatt, 2022; Wink & Sauer‐Gürth, 2000). Asian birds are sometimes referred to as the ‘Red‐headed Falcon’ (Naoroji, 2007; BirdLife International, 2024). Individuals from the Asian population are distributed from south‐east Iran, through Pakistan, India, Nepal and Bangladesh (del Hoyo et al., 2020). In Bangladesh, Red‐necked Falcons are difficult to observe in the field due to their small size (adult males 160 g vs. adult females 250 g; Tobias et al., 2022; Foysal & Panter, 2024) and are considered rare, localised breeders (Naoroji, 2007). The species inhabits open habitats interspersed with trees, including cultivated land and villages, but avoids dense forest and coastlines (Ali & Ripley, 1978; Bhatt, 2022; Cade & Digby, 1982; Dharmakumarsinhji, 1954; Foysal, 2014; Naoroji, 2007). Globally, the species is classified as near threatened by the International Union for the Conservation of Nature's Red List of Threatened Species (IUCN Red List) (BirdLife International, 2016; IUCN, 2023). Red‐necked Falcons are experiencing moderately rapid population declines predominantly driven by habitat degradation (Naoroji, 2007; Rasmussen & Anderton, 2005), with the number of mature individuals remaining unknown (BirdLife International, 2016).

Compared with their temperate conspecifics, tropical raptors are comparatively understudied (Buechley et al., 2019). Unlike similar, well‐studied species such as the Eurasian Kestrel (*F. tinnunculus*) and American Kestrel (*F. sparverius*) (McClure et al., 2023), the Red‐necked Falcon has been subject to a considerable lack of research attention. To date, most studies have concentrated on the species' breeding ecology (Foysal, 2014, 2015; Khan, 1978; Naoroji, 2011). Consequently, there is a paucity of basic understanding of this species' diet and how this varies in relation to human activities, with only a few studies having been conducted (Subramayana, 1985; Naoroji, 2011; Mahmood and Hussain, 2015; Pande et al. 2018; Bhatt, 2022; Foysal & Panter, 2024). Similar to other small‐ to medium‐sized raptors, Red‐necked Falcons tend to be specialist mid‐air hunters (Newton, 1979). A recent study by Foysal and Panter (2024) found that the species' diet in Bangladesh mainly comprised of birds and bats, with pronounced seasonal differences. Similar dietary compositions have been reported from Pakistan (Mahmood & Hussain, 2015). In India, Red‐necked Falcons tend to hunt nearly exclusively on birds (Bhatt, 2022; Naoroji, 2011) but have also been observed feeding on small mammals (Pande et al. 2018). Despite this, an outstanding knowledge gap persists in relation to how the species' diet varies in response to human activities especially in densely populated regions such as Bangladesh.

In this study, we leverage a long‐term data set comprising direct field observations of 15 Red‐necked Falcon pairs in Bangladesh between 2002 and 2019. We study the effects of climate and urbanisation on falcon diet, exploring relationships between mean monthly temperature (°C), precipitation (mm) and proportional urban land cover (%) on falcon prey weights and dietary composition. In a response to a call by Martínez‐Ruiz et al. (2023) for increased research on the effects of climate change on raptor diets, we also explore how mean monthly temperature differences (°C) affect falcon prey weights and dietary composition.

Given that the species exhibits pronounced seasonal differences in its diet (Foysal & Panter, 2024), we expect to find that falcons will hunt heavier prey, e.g., birds, during periods of the year with decreased temperatures and precipitation. In line with optimal foraging theory, we expect falcons to maximise fitness by hunting larger prey (providing the most benefit) for the lowest energetic expenditure costs (Sih & Christensen, 2001). Conversely, we expect to observe a shift in the falcon diet during periods of the year with increased temperatures and precipitation, i.e., during the rainy monsoon season when aerial insect abundances peak (Foysal & Panter, 2024), with a decrease in prey weight and an increase in the probabilities of bats. Assuming that urban areas are associated with increased artificial light at night (ALAN; Hopkins et al., 2018) and that ALAN increases predator activity (Bennie et al., 2018), we expect to find a positive relationship between the degree of urbanisation and the probability of bats in the diet as a result of increased ALAN in urban areas (Hopkins et al., 2018).

## 2 MATERIALS AND METHODS

### 2.1 Study area

Situated within subtropical south Asia, Bangladesh spans 20°34′ to 26°33′ North and 88°01′ to 92°41′ East. With a human population of approximately 170 million people, it is one of the most densely populated countries in the world (United Nations, 2022). Dhaka is the capital city of Bangladesh, located geographically in the centre of the country. Bangladesh spans the Indo‐Himalayas and Indo‐China major biotic subregions, and falls within the Oriental zoogeographical region (Khan, 2008). Seasonal variations in climate are marked by the tropical monsoon season occurring during June to October, followed by the cool dry winter period in November to February and the pre‐monsoon hot season between March and May (Foysal & Panter, 2024). Daytime temperatures range from 11 to 29°C in the cool dry winter and 21–34°C during the pre‐monsoon hot season (Foysal & Panter, 2024). Mean annual precipitation ranges from 1100 mm in the west to 5700 mm in the north‐east, with approximately 70%–80% falling during the rainy monsoon season (Khan, 2008).

### 2.2 Field observations

Direct field observations of 15 adult Red‐necked Falcon pairs feeding were conducted throughout the administrative divisions of Dhaka and Chittagong between 2002 and 2019. Field observations were performed by M.F. and began as early as 04:35 hrs (Bangladesh Standard Time/BST) typically ending at 10:00 h (BST), while later observations lasted from mid‐day up to 19:30 h (BST), approximately 30 min after sunset. Observations were conducted from the ground or a suitable rooftop using a Kowa TSN‐664 spotting scope 20–60x and 10 × 42 binoculars. In the field, prey species were identified to the lowest taxonomic level possible.

### 2.3 Taxonomic backbones and falcon prey data

All avian prey taxonomies were standardised according to the taxonomic backbone of BirdLife International which uses the taxonomy published by the Handbook of the Birds of the World and the BirdLife International Illustrated Checklist of the Birds of the World (Handbook of the Birds of the World and BirdLife International, 2020). We extracted species‐level mean prey weights (g) from the AVONET database (Tobias et al., 2022). For bats, we sourced mean prey weights from Wilmen et al. (2014) and Faurby et al. (2018). There were two observations of falcons feeding on invertebrates (unidentified Insecta sp. and Odonata sp.). Due to insufficient sample sizes, we omitted the invertebrate observations from the analyses. For full taxonomic methodological details, see Foysal and Panter (2024).

### 2.4 Land cover and climate data

We downloaded land cover data from the Global Land Cover 2000 database (Global Land Cover Database, 2003; https://forobs.jrc.ec.europa.eu/glc2000) on 10th April 2024. Land cover data were downloaded in raster file format at 1000 m resolution. Using QGIS version 3.14.16‐Pi (QGIS.org, 2024), we reclassified the land cover data using the ‘reclassify by table’ function to isolate only cells that contained the land cover type ‘artificial surfaces and associated areas’ (hereafter ‘urban land cover’). We created a new binary raster layer whereby all cells containing urban areas were assigned the value 1 and all other land cover types were reclassified a value of 0. No information has been published on the species' foraging range size. Therefore, we assigned 2 km circular buffers around each observation site, to reflect the average foraging range size of other similar small–medium‐sized falcons (Garrat et al., 2011; Village, 1982). Within these buffers, we extracted proportional urban land cover (%) values from the binary land cover raster layer.

We extracted climate data from the World Bank's Climate Change Knowledge Portal (https://climateknowledgeportal.worldbank.org/) on 9th April 2024. The following data variables were downloaded for 1991–2020: mean monthly surface air temperature (°C) (hereafter ‘mean temperature’), minimum monthly surface air temperature (°C), maximum monthly surface air temperature (°C) and mean monthly precipitation (mm). We omitted all climate data that did not coincide with our study period (2002–2019). To explore the effects of temperature fluctuations on Red‐necked Falcon diet, we created a new variable ‘mean temperature difference (°C)’ by calculating the difference between the minimum and maximum mean surface air temperature (°C) values for each month. We interpreted this variable as a proxy for potential human‐induced climate change, with greater mean monthly temperature differences representing expected future climatic patterns within the region (Kamruzzaman et al., 2023).

### 2.5 Statistical analyses

All statistical analyses were performed in R version 4.3.2 (R Core Team, 2023). We tested for collinearity between the mean temperature (°C), mean precipitation (mm), mean temperature difference (°C) and urban land cover (%) variables. Correlations whereby *r* ≥ .70 have been shown to inflate the variance of estimated regression coefficients (Dormann et al., 2013). We took a precautionary approach and considered all correlations *r* ≥ .60 to be colinear and thus dependent on one another, visualising these relationships using the ‘PerformanceAnalytics’ package (Peterson & Carl, 2020) (see Figures S1 and S2 for a visual representation). As such, we ran separate models to test the effects of each of the environmental predictor variables on Red‐necked Falcon prey weight and dietary composition.

To explore the effects of climate and urbanisation on Red‐necked Falcon prey weights, we ran a series of generalised linear mixed models (GLMMs) using the ‘lme4’ package (Bates et al. 2015). For all models, mean prey weight (g) was fitted as the response variable, with mean temperature (°C), mean precipitation (mm) and mean temperature difference (°C) fitted as explanatory variables across separate models. To account for non‐independence in the diet between pairs, we fitted ‘pair_id’ as a random effect. All prey weight models were fitted with ‘identity’ link functions and gaussian family error distributions. Type II ANOVAs were used to test for the significance of each individual explanatory variable. Conditional and marginal *R* ^*2*^ values were extracted using R package ‘performance’ (Lüdecke et al., 2021).

To examine the effects of climate and urbanisation on the probability of bats in the diet of Red‐necked Falcons, we created a binary response variable ‘is.bat’, scoring each feeding observation as either 1 or 0 depending on the presence or absence of bats as prey items, respectively. A series of GLMMs, were run with ‘is.bat’ fitted as the response variable and mean temperature (°C), mean precipitation (mm) and mean temperature difference (°C) fitted as explanatory variables across separate models. Again, we fitted ‘pair_id’ as a random effect and ran all bat models with ‘logit’ link functions and binomial error distributions.

## 3 RESULTS

**FIGURE 1:**
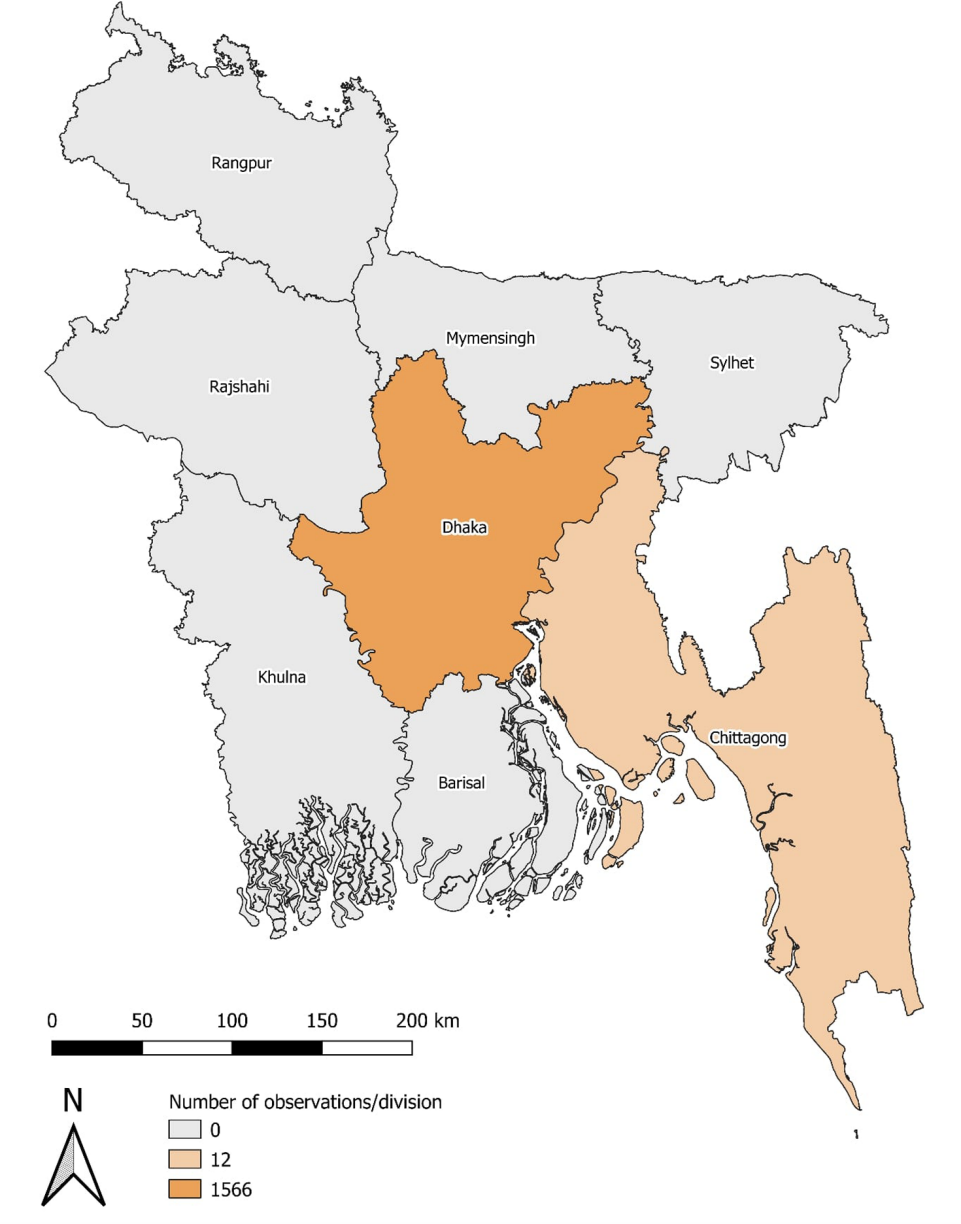
Spatial distribution of 15 Red‐necked Falcon (*Falco chicquera*) pairs observed in Bangladesh between 2002 and 2019. Map shows the distribution of 1578 observations of pairs feeding at the administrative division level.

Between 2002 and 2019, 1578 feeding events from 15 Red‐necked Falcon pairs were observed. Birds totalled 74.6% (*N* = 1177) of the diet and bats comprised 25.4% (*N* = 401) of the diet. House Sparrows (*Passer domesticus*) comprised the majority of the species' diet totalling 59.1% (*N* = 932) of all prey observations, followed by unidentified bird spp. (13.7%; *N* = 216). Other birds identified in the species' diet included the House Swift (*Apus nipalensis*) (0.8%; *N* = 12), unidentified swallow spp. (0.5%; *N* = 8), Coppersmith Barbet (*Megalaima haemacephala*) (0.1%; N = 2), Asian Palm Swift (*Cypsiurus balasiensis*) (0.1%; *N* = 2), Brown Shrike (*Lanius cristatus*) (0.1%; *N* = 1), Budgerigar (*Melopsittacus undulatus*) (0.1%; *N* = 1), Red‐vented Bulbul (*Pycnonotus cafer*) (0.1%; *N* = 1), Common Tailorbird (*Orthotomus sutorius*) (0.1%; *N* = 1) and unidentified starling spp. (0.1%; *N* = 1). Unidentified Chiroptera spp., most likely Indian Pipistrelles (*Pipistrellus coromandra*) totalled 25.3% (*N* = 400) of the species' diet, followed by the Greater Short‐nosed Fruit Bat (*Cynopterus sphinx*) (0.1%; N = 1). Field observations spanned two administrative divisions in Bangladesh with most being conducted in the division of Dhaka (*N* = 1566; 99.2%) followed by Chittagong (*N* = 12; 0.8%) (Figure 1). Across the field sites, mean proportional urban land cover was 62.3 ± 8.4% (0%–100%). Throughout the study period, the mean monthly temperature was 26.2 ± 3.5°C (± standard deviation [SD]; range: 17.2–29.6°C), mean monthly precipitation was 149 ± 141.3 mm (0.15–678.3 mm), with a mean monthly temperature difference of 10.3 ± 2.9°C (5–15.2°C) (Figure S2).

### 3.1 Climate and urbanisation effects on prey weight

**FIGURE 2:**
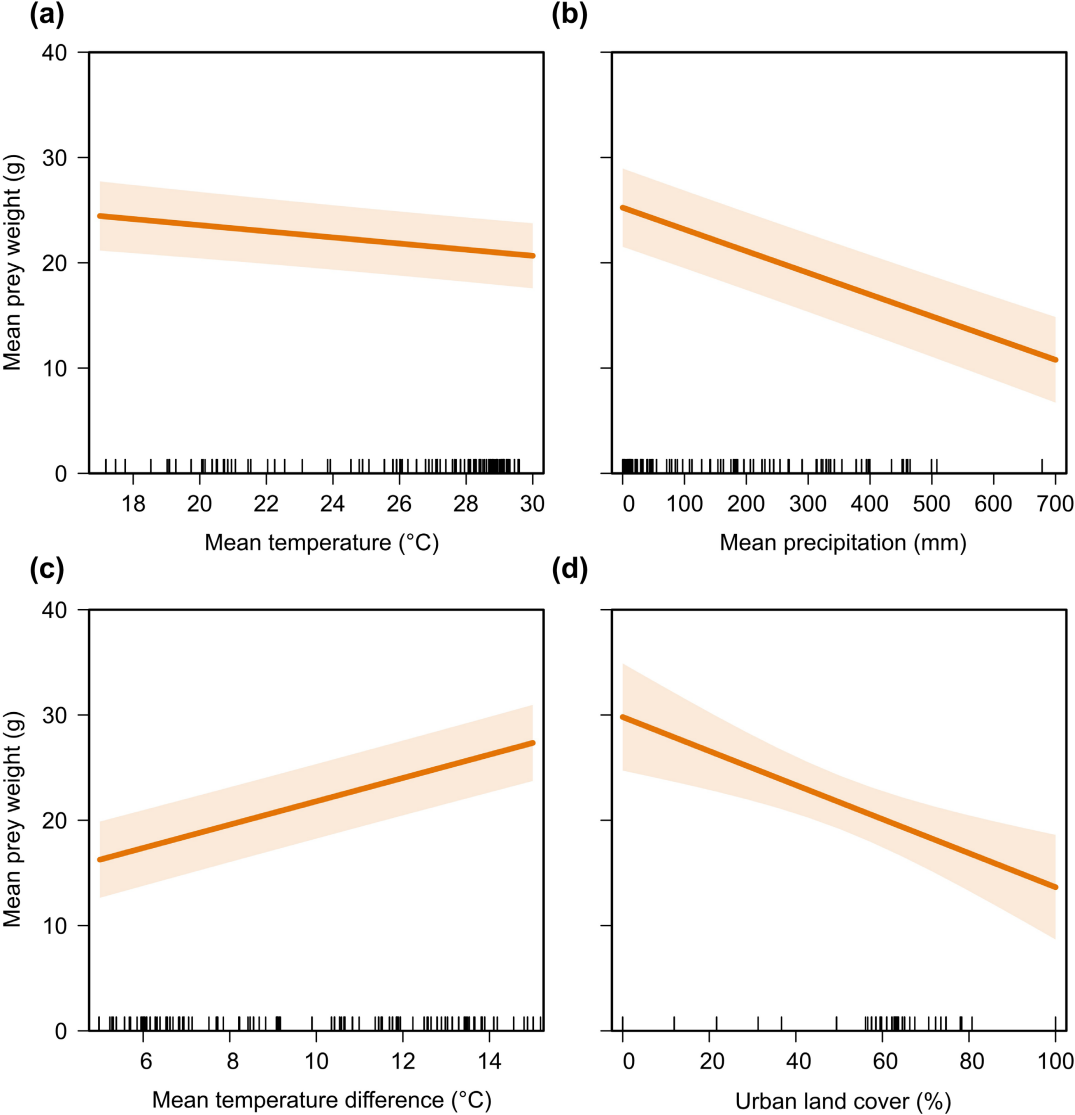
Modelled effects, with 95% confidence intervals, of (a) mean monthly temperature (°C), (b) mean monthly precipitation (mm), (c) mean monthly temperature difference (°C) and (d) urban land cover (%) on mean prey weights of 15 Red‐necked Falcon (*Falco chicquera*) pairs throughout Bangladesh between 2002 and 2019.

There were significant effects of mean temperature (*X* ^*2*^ = 18.21, *df* = 1, *p* < .0001; *R* ^*2*^ = .22), mean precipitation (*X* ^*2*^ = 161.96, *df* = 1, *p* < .0001; *R* ^*2*^ = .39), mean temperature difference (*X* ^*2*^ = 212.61, *df* = 1, *p* < .0001; *R* ^*2*^ = .39) and proportional urban land cover (*X* ^*2*^ = 13.32, *df* = 1, *p* = .0002; *R* ^*2*^ = .15) on mean prey weights of Red‐necked Falcons (Table 1). Specifically, falcons preyed on small prey items during months with higher temperatures (Figure 2a), months with higher precipitation (Figure 2b) and in more urban areas (Figure 2d; Table 1). Conversely, there was a significant positive effect of mean temperature difference, with falcons preying on heavier prey items during months with higher temperature differences (Figure 2c; Table 1).

**TABLE 1:** Model estimates exploring the effects of mean monthly temperature (°C), mean monthly precipitation (mm), mean monthly temperature difference (°C) and urban land cover (%) on mean prey weights (g) of 15 Red‐necked Falcon (*Falco chicquera*) pairs in Bangladesh between 2002 and 2019.

Variable	Estimate	SE	*Df*	*t*	*p*	*R* ^*2*^
Temperature						
(Intercept)	29.3860	2.3653	31	12.424	<.0001	.22
mean temperature (°C)	−0.2907	0.0681	1575	−4.268	<.0001	
Precipitation						
(Intercept)	25.2400	1.8940	10	13.320	<.0001	.39
Mean precipitation (mm)	−0.0207	0.0016	1576	−12.730	<.0001	
Temperature difference						
(Intercept)	10.7200	1.9620	13	5.462	<.001	.39
Mean temperature difference (°C)	1.1090	0.0760	1572	14.581	<.0001	
Urban land cover						
(Intercept)	29.8073	2.5938	27	11.490	<.0001	.15
Urban land cover (%)	−0.1616	0.0443	22	−3.650	.001	

Abbreviations: *df*, degrees of freedom; SE, standard error.

### 3.2 Climate and urbanisation effects on dietary composition

**FIGURE 3:**
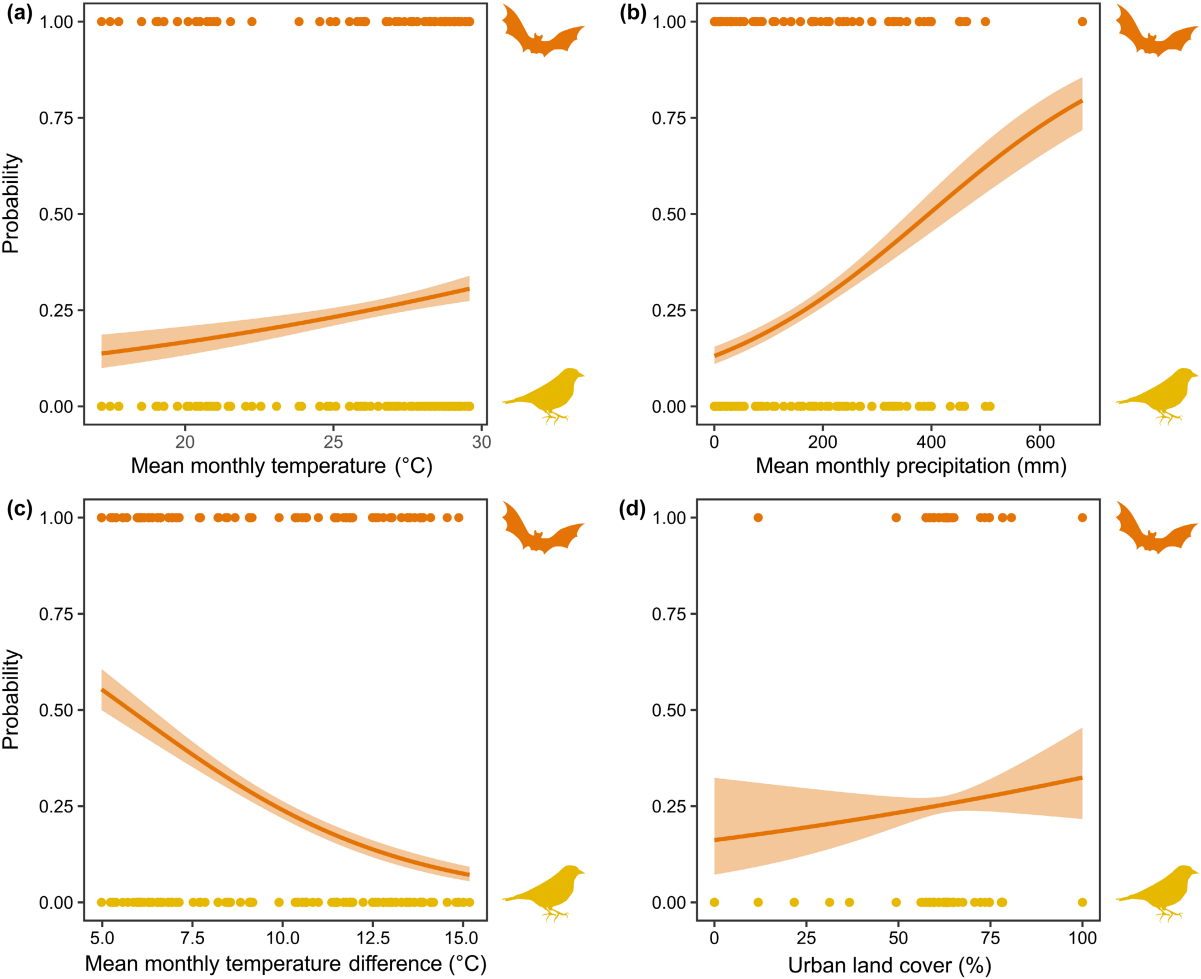
Probabilities, with 95% confidence intervals, of bats and birds in the diets of 15 Red‐necked Falcon (*Falco chicquera*) pairs in relation to (a) mean monthly temperature (°C), (b) mean monthly precipitation (mm), (c) mean monthly temperature difference (°C) and (d) urban land cover (%) in Bangladesh between 2002 and 2019.

There were significant effects of mean temperature on the dietary composition of Red‐necked Falcons (*X* ^*2*^ = 19.64, *df* = 1, *p* < .0001), with falcons being significantly more likely to prey on bats during warmer months (Figure 3a; Table 2). This effect was the same for mean monthly precipitation (*X* ^*2*^ = 123.77, *df* = 1, *p* < .0001), with a decreased probability of bats in the diet during wetter months (Figure 3b; Table 2). Conversely, mean temperature difference had a significant effect on the dietary composition of Red‐necked Falcons (*X* ^*2*^ = 152.49, *df* = 1, *p* < .0001), whereby months with greater temperature differences had fewer bats in the falcon diet (Figure 3c; Table 2). There was also a significant effect of urban land cover on the probability of bats in the falcon diet (*X* ^*2*^ = 5.06, *df* = 1, *p* = .245), with pairs from more urban areas being significantly more likely to prey on bats than those from more rural areas (Figure 3d; Table 2).

**TABLE 2:** Model estimates exploring the effects of mean monthly temperature (°C), mean monthly precipitation (mm), mean monthly temperature difference (°C) and urban land cover (%) on the probability of bats in the diet of 15 Red‐necked Falcon (*Falco chicquera*) pairs in Bangladesh between 2002 and 2019.

Variable	Estimate	SE	*Df*	*z*	*p*	*R* ^*2*^
Temperature						
(Intercept)	−3.2475	0.4978	1577	−6.524	<.0001	.02
Mean temperature (°C)	0.0820	0.0185	–	4.432	<.0001	
Precipitation						
(Intercept)	−2.0553	0.2162	1577	−9.506	<.0001	.17
Mean precipitation (mm)	0.0051	0.0005	–	11.125	<.0001	
Temperature difference						
(Intercept)	1.6319	0.2969	1577	5.496	<.0001	.23
Mean temperature difference (°C)	−0.2885	0.0234	–	−12.349	<.0001	
Urban land cover						
(Intercept)	−3.4960	1.0693	1577	−3.270	.001	.12
Urban land cover (%)	0.0381	0.0169	–	2.249	.025	

Abbreviations: *df*, degrees of freedom; SE, standard error.

## 4 DISCUSSION

Our study represents the first to explore the effects of climate and urbanisation on the diet of the globally near threatened and declining Red‐necked Falcon in Bangladesh. Falcons were more likely to hunt smaller prey during warmer and wetter months, and also in more urban areas. Red‐necked Falcons are specialised aerial hunters and these dietary patterns were mediated by the probability of bats and birds within the species' diet. Consumption of bats, especially during twilight hours, is common in small‐sized aerial raptors which also include other falcons such as the Eurasian Hobby (*F. subbuteo*) and Amur Falcon (*F. amurensis*) (Feng et al., 2022; Stanton, 2016). Building on previous research focusing on the species' diet throughout its Indian subcontinent range (Subramayana, 1985; Naoroji, 2011; Mahmood and Hussain, 2015; Pande et al. 2018; Bhatt, 2022; Foysal & Panter, 2024), we improve our understanding of the trophic ecology of this considerably understudied subtropical raptor.

### 4.1 Warmer and wetter months may increase insect availability

As expected, falcons hunted smaller prey, i.e., bats, during months with increased temperatures and precipitation rates. Recent research found that that insectivorous *Pipistrellus* spp. bats, most likely Indian Pipistrelles, comprised the majority of all chiropteran prey of Red‐necked Falcons in Bangladesh during the rainy monsoon season (Foysal & Panter, 2024). It is well‐documented that insect populations vary with climatic conditions, whereby lower temperatures inhibit population growth (Anthony et al., 1981; Burles et al., 2009). Increased precipitation rates also allow dipteran and lepidopteran populations to multiply rapidly (Debata et al., 2019; Frick et al., 2009), which are major food sources for insectivorous bats. Changes in temperature and precipitation also influence flowering and fruiting of food plants, which impacts the foraging behaviours of frugivorous and nectivorous bats (Richter & Cummings, 2008). Optimal foraging theory implies that animals should maximise fitness by providing themselves with the most energetic benefit in return for the lowest energetic expenditure cost (Sih & Christensen, 2001). Therefore, we expected to find an increase in birds within the diet during periods of low resource availability, i.e., during drier and colder months. We observed a negative relationship between temperature and precipitation on falcon prey weight, i.e., falcons hunted larger prey during colder and drier months, maximising the rewards obtained from exerting energy expenditure while hunting during periods of low resource availability.

### 4.2 Artificial light at night in urban areas can increase foraging opportunities

Urban falcon pairs were more likely to prey on bats, whereas more rural pairs tended to prey on birds. This pattern was also evident in the prey weight analysis, with more urban pairs feeding on smaller prey items. Urban areas are associated with increased ALAN (Hopkins et al., 2018) and provide extended hunting opportunities for predators beyond daylight hours (Bennie et al., 2018). Avian predators, including Red‐necked Falcons, may benefit from increased ALAN which attracts invertebrate prey and improves visibility during hunting activities at night (Rodríguez et al., 2020). This increased hunting opportunity, coupled with suitable environments for bats within urban areas in Bangladesh, e.g., presence of roosting sites in large trees and crevices between old buildings (Aziz et al., 2007), may explain why we detected an increase in bats within the diet of urban falcon pairs. Furthermore, observational evidence from Bangladesh suggests that Indian Pipistrelles are frequently observed at night flying close to streetlights where they feed on nocturnal insects (Aziz et al., 2007). However, many bat species actively avoid artificial lighting (Seewagen & Adams, 2021), thus our dietary analysis may only reflect a proportion of the bat fauna consumed by Red‐necked Falcons. Given this, global urbanisation rates are expected to increase in the future, especially in Bangladesh which is one of the most densely populated countries in the world (United Nations, 2022). Wildlife will therefore have to adapt to human‐induced changes to the natural environment. Our approach did not allow us to directly test the effects of ALAN on falcon diet; but future research should consider including this as a potential influential predictor.

### 4.3 Climate change has the potential to both negatively and positively affect raptors with specialised feeding strategies

Mean monthly temperature difference, i.e., our proxy for climate change, was better at explaining the modelled probabilities of bats and birds in the falcon diet compared with mean temperature alone (*R* ^*2*^ mean temperature difference = 21% vs. *R* ^*2*^ mean temperature = 2%). Our data suggested that during months with increased temperature differences, falcons appeared to shift their dietary niche towards larger prey such as birds. Conversely, there was a substantial decrease in the probability of bats in the diet during more variable months. This may be explained by nighttime temperatures being colder, reducing insect activity and bat activity with fewer opportunities for falcons to prey on bats. Falcons may adapt their diet according to climatic conditions, for example, by preying on fewer bats in months with greater mean temperature differences relative to birds. As a consequence of human‐induced climate change, extreme shifts in seasonal temperature fluctuations are expected to increase throughout the Indian subcontinent in the future (Pal & Al‐Tabbaa, 2009). Our findings suggest that climatic changes may impact the trophic ecology of predator species such as the Red‐necked Falcon, however not always necessarily in a negative way. Shifts in the prey available to particular raptor species may allow them to expand their dietary niche in response to environmental changes. Alternatively, due to their narrow ecological niches and lack of plasticity, raptors with specialised habitat or feeding strategies, such as aerial hunters, are at risk of being disproportionately affected by climate change (Gilg et al., 2012; Hof et al., 2012). Future research should focus on elucidating the impacts of ongoing human‐induced climate change and study how predatory species adapt to changing environments.

### 4.4 Study limitations

Despite being the first to study the effects of climate and urbanisation on the diet of Red‐necked Falcons in Bangladesh, our study is not without limitations. Most notably, the explanatory power of our models remained relatively low (mean prey weight model *R* ^*2*^ range: 15–39%; bat models: 2%–23%), suggesting that a substantial proportion of heterogeneity remained unaccounted for by our climatic and urbanisation explanatory variables. Our large sample size may explain why some of these patterns returned statistical significance, but it should be noted that such patterns may not actually reflect biological significance and thus inference from models with low *R* ^*2*^ values, i.e., ~2%, should be made with caution. Similarly, we ran separate models due to collinearity between predictor variables (*r* ≥ .60), however, repeat testing of these variables may increase the probability of Type I error (Underwood, 1997). To avoid this, future research should endeavour to explore the effects of more influential predictors of falcon diet, including intrinsic (e.g., sex and age) and extrinsic factors (e.g., prey availability, ALAN, human density). Leveraging a long‐term data set comprising direct field observations may introduce a prey‐size bias towards larger, more obvious prey items. Observations were often made from a roof top and we cannot account for potential misidentifications of prey items. Coupling these data in combination with other approaches to study raptor diets will likely greatly improve the accuracy of our dietary estimates. For example, analysing pellets and prey remains (Lewis et al., 2010; Redpath et al., 2001), DNA and metabarcoding techniques (Bourbour et al., 2019, 2021, 2024; Hacker et al., 2021; Nota et al., 2019), web‐sourced photography (Kannan et al., 2023; Naude et al., 2019; Panter & Amar, 2021, 2022) or stable isotope analyses (Catry et al., 2016; Johnson et al., 2020; Jones et al., 2024), can be used in combination with direct observations from the field to improve estimates of raptor diets.

## 5 CONCLUSIONS

Our findings provide the first quantitative assessment of the impacts of climate and urbanisation on an understudied and globally near threatened subtropical raptor. Falcons responded to changes in their environment by shifting their dietary niches from bats to birds depending on climate and the degree of urbanisation. However, our models contained substantial unaccounted heterogeneity which our explanatory variables failed to explain. Therefore, future research should focus on unpicking relationships between falcon diet and other influential factors such as sex, age, prey availability, human densities and ALAN. Given that rates of urbanisation and human‐induced climate change are expected to continue into the foreseeable future, their effects on wildlife need to be better understood. This study represents an initial advance in our understanding of how falcons respond to an increasingly human‐dominated world.

## AUTHOR CONTRIBUTIONS

**Mohammod Foysal:** Conceptualization (equal); data curation (equal); methodology (equal); writing – review and editing (equal). **Connor T. Panter:** Conceptualization (equal); formal analysis (equal); investigation (equal); project administration (equal); supervision (equal); validation (equal); visualization (equal); writing – original draft (equal).

## Supporting information


Figures S1–S2.

## Data Availability

Data, code and readme files associated with this study are available via Figshare (see https://doi.org/10.6084/m9.figshare.26661463.v1; https://doi.org/10.6084/m9.figshare.26869156.v1; https://doi.org/10.6084/m9.figshare.26869183.v1).
