# Supplementary material for: Synergistic effects of climate and urbanisation on the diet of a globally near threatened subtropical falcon
Source: Ecol Evol. 2024 Sep 10;14(9):e70290. doi: 10.1002/ece3.70290 (PMC11387113; doi:10.1002/ece3.70290)
Supplement: Supplementary file 1 — Figures S1–S2. [file ECE3-14-e70290-s001.docx]

Supporting information for “*Synergistic effects of climate and urbanisation on the diet of a globally near threatened subtropical falcon*”

**
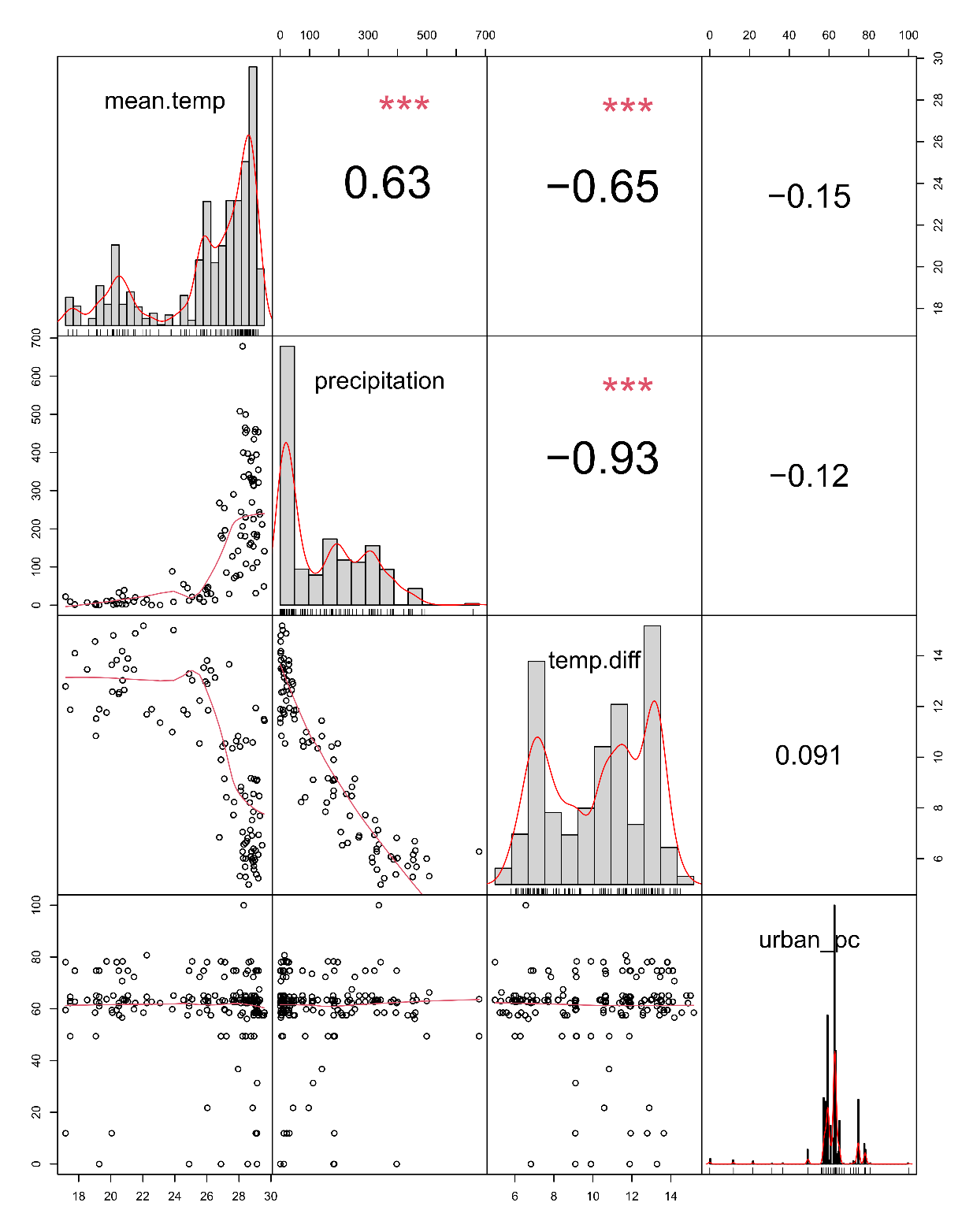
**

**Figure S1.** Output from the test for collinearity between explanatory variables: mean temperature (°C), mean precipitation (mm), mean temperature difference (°C) and urban land cover (%) on Red-necked Falcon diet between 2002 and 2019.

**
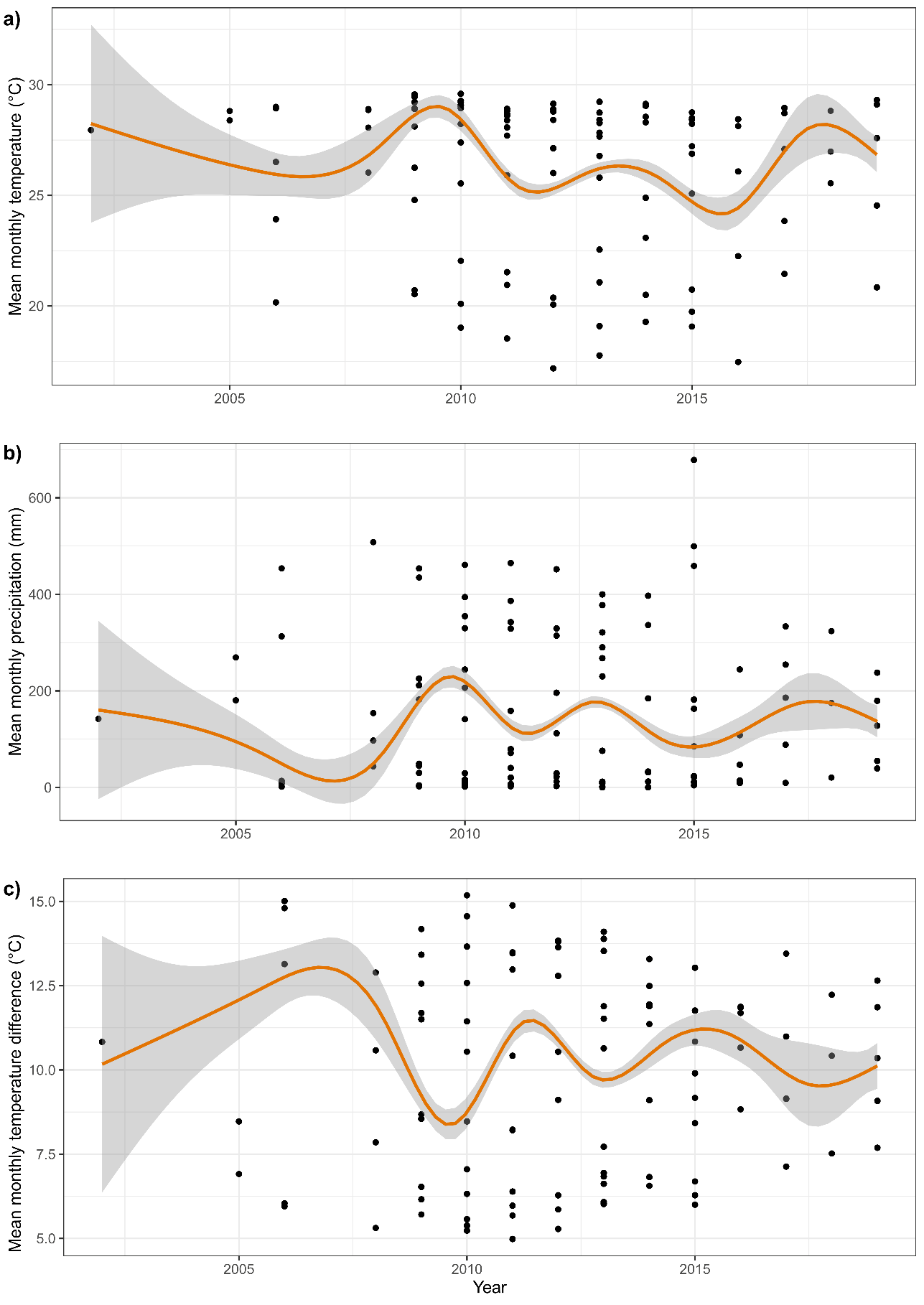
**

**Figure S2.** Trends over time in a) mean monthly temperature (°C), b) mean monthly precipitation (mm) and c) mean monthly temperature difference (°C) in Bangladesh between 2002 and 2019. Line of best fit depicts a GAM smoothing operator with 95% confidence intervals for visualisation purposes only.
